# Disciplinary differences in the experience of online education among teachers and students in Chinese universities during COVID-19

**DOI:** 10.3389/fpsyg.2022.909269

**Published:** 2022-09-14

**Authors:** Shuo Yu, Ying Liu, Bingqing Yang, Zhiwei Chen

**Affiliations:** ^1^College of International Education, Minzu University of China, Beijing, China; ^2^School of Education, Zhengzhou University, Zhengzhou, China; ^3^School of Education, Minzu University of China, Beijing, China

**Keywords:** online education, academic disciplines, teachers, students, COVID-19

## Abstract

Online education has advantages during COVID-19, but it also has problems related to hardware support and user experience. Focusing on teaching quality by discipline is an effective way to improve teaching quality in universities. To investigate the online education experience from the perspective of different academic disciplines, we evaluated 251,929 student questionnaires and 13,695 teacher questionnaires from 334 universities in China. The main finding was a difference in teaching preparation, experience, feedback, and improvement processes by disciplines. Teachers and students had obvious disciplinary differences in preparation, school support, and teaching constraints. However, disciplinary differences were minor for pedagogical issues such as participation, assignments, and grading, as well as for evaluation of platform technical support and views on the continuation of online learning. The research results analyzed the teaching psychology of teachers and students in different disciplines during the pandemic. Therefore, it explained the impact and role of discipline differences on students’ learning psychology during COVID-19. This research will benefit educators, researchers, and policy makers to help them with the improvement of online education.

## Introduction

As one of the first countries in the world to be affected by COVID-19, China deployed an online education platform on January 30, 2020 (Ministry of Education, 2020). Since then, many online education platforms independently developed by China, such as Rain Classroom, China University Massive Open Online Courses (MOOCs), ZOOM, Zhihuishu, and Chaoxing Erya, have begun to be used on a large scale.

Online education has advantages during pandemic, but it also has problems related to hardware support and user experience (Hu and Xie, 2020). Therefore, university administrators, educational experts, and parents have had doubts about the quality of online education. Moreover, based on China’s current conditions, finding ways to improve the quality of teaching in colleges and universities is a top educational issue. Since the level and popularity of a university depends on the various disciplines found within it, focusing on teaching quality by disciplines is an effective way to improve teaching quality in universities. When comparing different disciplines, significant differences have been found in Chinese college students’ evaluations related to teaching mode, interaction, teaching process, assessment process, and learning experience (Chen and Jia, 2020). The differences reflect the different cognitive approaches of university students from different disciplinary backgrounds in various dimensions. From a deeper perspective, these differences reflect an unbalanced development of online education in different disciplines and the different depth of teaching advancement among disciplines.

The popularization of online education has been inevitable in the face of the education informatization 3.0 (Shang et al., 2016). As students of different disciplines have different understandings of teaching concepts and thus different learning effects (Wood and Solomonides, 2009; Parpala et al., 2011), it is recommended that universities can make good use of this large-scale online education practice and explore the differences between disciplines from the perspective of both teachers and students. Therefore, this research focused on two questions: (1) Does the online education experience of teachers and students in colleges and universities differ in regard to teaching preparation, experience, feedback, and improvement processes? (2) What are the specific differences between teachers and students of different disciplines in those four processes?

## Literature review and research questions

### Classification and characteristic of disciplines

Knowledge is divided according to certain logic, with the establishment of identities and boundaries. On this basis, a community of academics will be formed with division of labor (Yan, 2008). Therefore, inherent differences in the classification and characteristics of disciplines relate to differences of the knowledge systems. At first, based on the concept of “paradigm,” Kuhn (1970) divided disciplines into “high paradigm disciplines” and “low paradigm disciplines.” High paradigm disciplines, such as biology, chemistry, and physics, have reached a high level of unity in the theoretical system and value norms, reflecting consistency of teachers’ views on the discipline’s theories, methods, skills, and other issues. In contrast, low paradigm disciplines, such as history, pedagogy, psychology, and sociology, have inconsistent knowledge bases and research modes. In addition, Biglan (1973) summarized three dimensions for the division of academic fields through empirical research: disciplinary paradigm (hard science vs. soft science), attention to the degree of application (pure science vs. applied science), and difference between the natural world and human society (life science vs. other science), further highlighting the diversity of the disciplines’ epistemological characteristics. Later, Kolb (1981) classified learning styles from the two perspectives of “Abstract conceptualization ↔ Concrete experience” and “Active experimentation ↔ Reflective observation.” In this way, disciplines were classified into four types: abstract conceptualization–theoretical discussion (pure hard science), abstract conceptualization–active experimentation (applied hard science), concrete experience–active experimentation (applied soft science), and concrete experience–theoretical research (pure soft science). Similarly, Becher and Trowler (2008) divided disciplines into pure hard science, pure soft science, applied hard science, and applied soft science. Different categories of disciplines require spaces to support concentration or interaction (Huhtelin and Nenonen, 2019). Studying the knowledge characteristics and cultural differences of various disciplines allows analysis of the interactive relationship between discipline epistemology and discipline culture. Different disciplinary classifications can even influence bibliometric findings (Sīle et al., 2019). It is also mentioned that the diversity of disciplines leads to different ways of teaching, which also shows that the logical differences within the disciplines inevitably cause external practice differences (Hartwell et al., 2017).

### Practical differences among disciplines

The practical differences of the disciplines are manifested in different goals, concepts, and social values (Kekäle, 2000). Furthermore, Moses (1990) found that the humanities and social sciences have advantages in acquiring and expanding library resources, while chemistry and engineering are stronger in obtaining funds. The performance indicators used by institutions focus on quantifiable processes and external results, which is unfair for the humanities and social sciences and more supportive of physics and applied sciences. Compared with scholars in the hard sciences, scholars in the humanities publish works fewer but longer.

Differences are also reflected in the teaching process and training results. Studies in the past few years have confirmed that students in different disciplines show certain differences in learning stages. Marquis and Vajoczki (2012) believed that there were differences in intrinsic study motivation for different disciplines, and the motivation from disciplines can play a role in improving academic performance. Disciplines will directly influence the perceived quality of professors’ teaching effectiveness (Chiu et al., 2019), as well as student evaluations of teaching (Wolfgang, 2020). Moreover, scientific performance and collaboration also differ from different disciplines (Rojko and Lužar, 2022); such as the doctoral supervisors’ supervisory activities and intentions vary much by discipline (Kreber and Wealer, 2021).

Xie and Ma (2013) found that humanities students are better than science and medical students in classroom learning habits, learning initiative, enthusiasm, and learning efficiency, while medical students are more proactive in acquiring new knowledge. Moreover, Wood and Solomonides (2009) and Parpala et al. (2011) believed that students of different disciplines have different understandings of teaching concepts and different attitudes toward teaching evaluation, leading to differences in learning effects and course evaluations. Teachers have differences in teaching mode and pedagogical approaches among different disciplines as well. Lin (2012) found that teaching modes differ by discipline due to the different requirements of science and engineering students and liberal arts students for teachers and teaching methods. Similarly, from the perspective of pedagogical approaches, Chen et al. (2012) believed that liberal arts students, who pay more attention to social realistic problems, show more positive performance compared with science and medical students. However, students in disciplines with practical courses, such as science, engineering, and medicine, have more opportunities for actual practice than those in disciplines with theoretical courses, such as liberal arts or social sciences (Liu, 2019).

In terms of training results, researchers have explored both learning effects and employment. Berdie (1967) found differences in the academic achievement of students from different disciplines. A subsequent study showed that students can gain a lot from their discipline, including social benefits brought by the discipline (Sanders-Dewey and Zaleski, 2009). In this regard, a study by Angoff and Johnson (1990) on the changes in students’ abilities in different disciplines before and after enrollment further confirmed these views. Employment is the most important outcome when examining the quality of talent cultivation in colleges and universities. Studies have also noted that the labor market has different demands for talents, leading to significant differences in the employment situation of college graduates in different disciplines and majors (Bee and Dolton, 1990; Woodley and Brennan, 2000).

### Online education research during the pandemic

Although scholars have conducted extensive research on interdisciplinary differences related to teaching, the studies have been based on the traditional in-person teaching mode. In the face of the massive online education required by COVID-19, how is online education going in universities? Are there any differences among disciplines? How do the differences occur? Since the beginning of 2020, many scholars have explored different perspectives of these questions.

In the context of COVID-19, students need to deal with various sorts of ecological, electronic, and mental battles (Garg et al., 2020). It is found that the information communication technology (ICT) is making teaching and learning attractive and useful during COVID-19 (Kumar et al., 2020). Compared with the online education in general, the “Emergency Remote Teaching” (ERT) has been mentioned with the characters of shifting face to face courses to online delivery modes, in order to provide instruction during a crisis situation (Affouneh et al., 2020; Bozkurt and Sharma, 2020; Hodges et al., 2020). Although it has been used in other countries before 2020 (Davies and Bentrovato, 2011), ERT can still create motivational factors that might circumvent some of the negatives associated with online education as it emerged again out of this pandemic crisis (Bawa, 2021). With ERT, universities can therefore achieve the transition from a “traditional teaching mode” and “online teaching mode” to an “online and offline blended teaching mode” (Qiu and Li, 2020). In the context of ERT, online teaching has a whole new meaning for both students and teachers. Therefore, different performances and attitudes toward online learning will be formed according to different factors, such as disciplines.

As to the situation in China, from a macro point of view, some scholars combined their own experience to trace the evolutionary path of China’s higher education technology in the past 40 years and discussed the dilemma of promoting education technology in China (Wu, 2020). Some scholars used macro data to describe and analyze the advantages, challenges, and highlights of online education in response to COVID-19 (Hu and Xie, 2020; Liu, 2020). Still other scholars analyzed the online teaching practice experience of three foreign universities for an international comparison (Xue and Li, 2020).

From a micro point of view, scholars have also examined the experiences and outcomes of students and teachers. Jia et al. (2020) studied the online learning experience of college students through cluster analysis. Zheng et al. (2020) used the ANOVA method to explore the online education attitudes of college teachers with different backgrounds in the post-COVID-19 era. Some researchers explored the teaching situation in relation to disciplinary characteristics to predict the development of online education in the future. Still other scholars focused on differences between regions in China and different universities, examining the online education experience of teachers and students by using descriptive statistics and difference analysis (Guo et al., 2020; Wu et al., 2020). In addition, based on above studies, some scholars evaluated the factors that influence college students’ online learning effectiveness, satisfaction, and learning engagement by using regression analysis and structural equation modeling (Rao and Wan, 2020; Shen and Wu, 2020).

In this effort, some studies found disciplinary differences in online education processes. For example, Zheng et al. (2020) found that teachers and students of different disciplines had different degrees of willingness to improve online education. At the same time, Wu et al. (2020) found that teachers of science, engineering, agriculture, and medicine carried out more online education than teachers in the liberal arts and social sciences before the outbreak of COVID-19. Wu and Li (2020) have mentioned that the statistical analysis method of scoring items as interval data to calculate the average value can be used to investigate students’ online learning performance in different disciplines. In this way, this research used the statistical analysis method to calculate the average value to do the analysis of questionnaire data.

### Summary and evaluation of research literature

There is a long history of studies, both domestically and internationally, on differences among disciplines. However, most research has been based on traditional teaching methods, with few studies from the perspective of online education. With the large-scale online education during the current pandemic, researchers have explored the macro teaching situation, micro classroom situation, and influencing factors, and most have applied quantitative analysis. However, few studies have addressed the online education situation from the perspective of disciplinary differences, and most existing research has been based on the analysis of differences between regions and colleges. Even though some studies have mentioned differences by disciplines, their results were relatively scattered and lacked verification. Moreover, the research subjects were either students or teachers; very few studies have explored the differences between teachers’ and students’ online education experience considering many educational dimensions. The current study begins to fill this research gap.

### Research questions

Based on the literature review, we propose the following research questions:

Question 1: Are there disciplinary differences between teachers and students in colleges and universities? If yes, how many dimensions of these differences?
Question 2: Will teachers and students of five disciplines groups have different levels of proficiency in the online education technology?
Question 3: Is there significant disciplinary difference in the choice of teaching mode between teachers and students in colleges and universities.
Question 4: Are there significant disciplinary differences in the teaching process, such as classroom discussion, teaching effectiveness, etc.?
Question 5: Is there significant disciplinary difference related to the attitudes of teachers and students toward the future of online education.

## Materials and Methods

### Sample selection

The study sample comprised teachers and students in Chinese colleges and universities who used online education during the COVID-19 period. A total of 13,997 surveys were sent to teachers and 256,504 to students in 334 colleges and universities in China through an online platform. In the end, as shown in Table 1, 13,695 valid questionnaires for teachers (43.3% male and 56.7% female) and 251,929 valid questionnaires for students (43% male and 57% female) were collected. The effective response rates were 97.8 and 98.2%, respectively. The proportion of teachers and students in different disciplines was as follows: philosophy, 1.7% teachers and 0.4% students; economics, 4.8% teachers and 8.2% students; law, 4.0% teachers and 2.9% students; pedagogy, 7.6% teachers and 6.6% students; literature, 12.6% teachers and 9.7% students; history, 1.0% teachers and 0.5% students; science, 11.8% teachers and 11.5% students; engineering, 29.4% teachers and 29.8% students; agriculture, 2.7% teachers and 2.1% students; medicine, 3.2% teachers and 4.3% students; management, 11.0% teachers and 13.5% students; and art, 10.2% teachers and 10.5% students.

**Table 1:** Socio-demographic characteristics of the sample.

Factors	Gender		Discipline				
Categories	Male	Female	Literature	Social Science	Science	Engineering	Medicine
Teachers	43.3%	56.7%	25.5%	27.4%	11.8%	32.1%	3.2%
Students	43%	57%	21.1%	31.2%	11.5%	31.9%	4.3%

### Survey design

This study is based on the “Online Teaching Survey” conducted by the Teacher Development Center of Xiamen University from March 13 to March 31, 2020. The questionnaire had four parts: basic information, online education environment and support, online education experience, and improvement for online education. In the basic information section, respondents could choose from among 12 disciplines based on China’s “Undergraduate Discipline Catalog of General Colleges and Universities,” namely, economics, art, science, literature, engineering, pedagogy, medicine, management, history, philosophy, agriculture, and law. The disciplines were integrated into five groups: humanities (including literature, philosophy, history, and art), social sciences (including economics, pedagogy, law, and management), science, engineering (including engineering and agriculture), and medicine (Yi, 2015). The other three parts of the survey addressed online education tools; online education service reliability; personal preparation; online education evaluation; online education influencing factors; problems, difficulties, and challenges; and improvements in online education. The online education experience of teachers and students was divided based on the stages of the teaching process: teaching preparation, teaching experience, teaching feedback, and teaching improvement. The questionnaire comprehensively reflected the relevant information on online education, which made it suitable for this research.

All survey questions used a five-point Likert-type scale from 1 to 5. For example, in the question “How familiar are you with the technology of various online teaching platforms?” the options were 1, very unskilled, 2, not skilled, 3, normally skilled, 4, skilled, and 5, very skilled. There were two exceptions, where the options were either yes (assigned a 2) or no (assigned a 1). These questions asked about the development of online education before and after COVID-19 and whether teachers and students had received relevant training. All items were scored as interval data to calculate the average value (Wu and Li, 2020).

The research screened out four dimensions, including 15 items, namely technical support (five items), online skills (four items), teaching strategies (four items), and tutoring (two items). The coefficients of the four dimensions based on the Cronbach’s alpha were 0.91, 0.83, 0.86, and 0.78, respectively. The reliability coefficients of each dimension were all higher than 0.7, indicating that reliability of the questionnaire is good. Factor analysis was used to explore the construct validity of this questionnaire. The cumulative variance contribution rate was 81.3%, and the coincidence of each factor was between 0.62 and 0.91, indicating good validity exists (Shen and Wu, 2020). The above analysis results showed that the validity and reliability of the questionnaire were good, and it was suitable for research on the online teaching experience of teachers and students in universities.

## Results

### Disciplinary differences in teaching preparation

#### Online education before and after COVID-19

**Figure 1:**
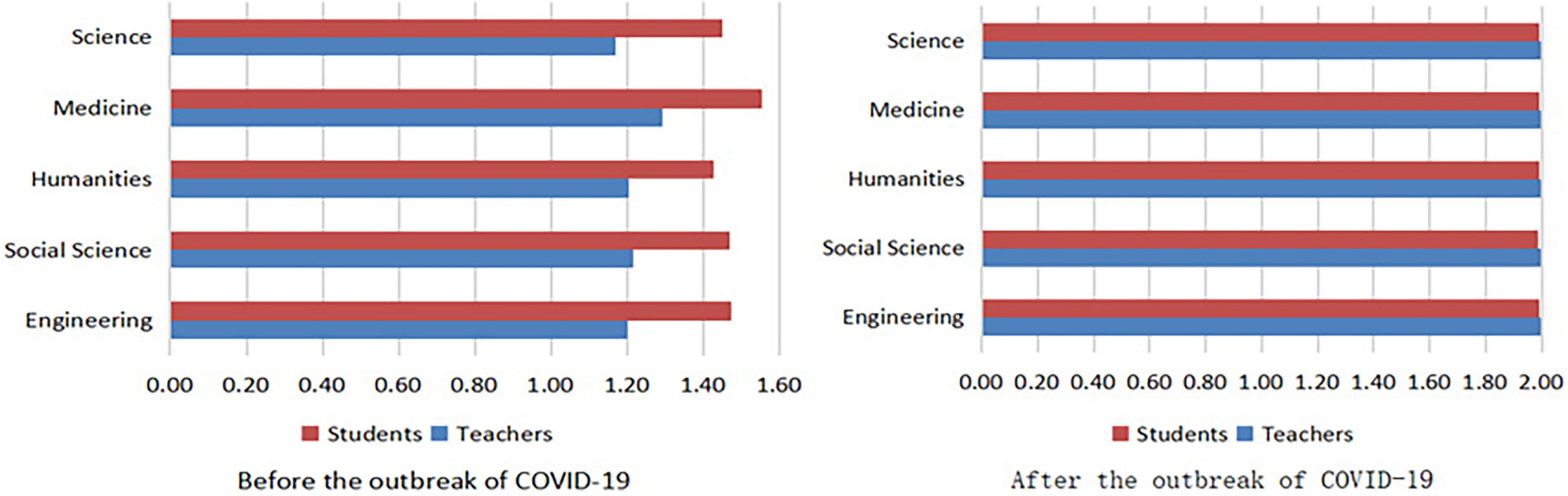
Implementation of online learning reported by teachers and students from different disciplines before and after the outbreak of COVID-19.

As shown in Figure 1, after the COVID-19 outbreak, the number of teachers and students of different disciplines participating in online education increased significantly. This indicates that the policy of “suspended class, ongoing learning” during COVID-19 was well implemented. Among the disciplines, medicine had the most use of online education before COVID-19, adding to teachers’ and students’ practical experience with it. After the outbreak, teachers and students in all disciplines had equal participation in online learning.

#### Training experience and proficiency

**Figure 2:**
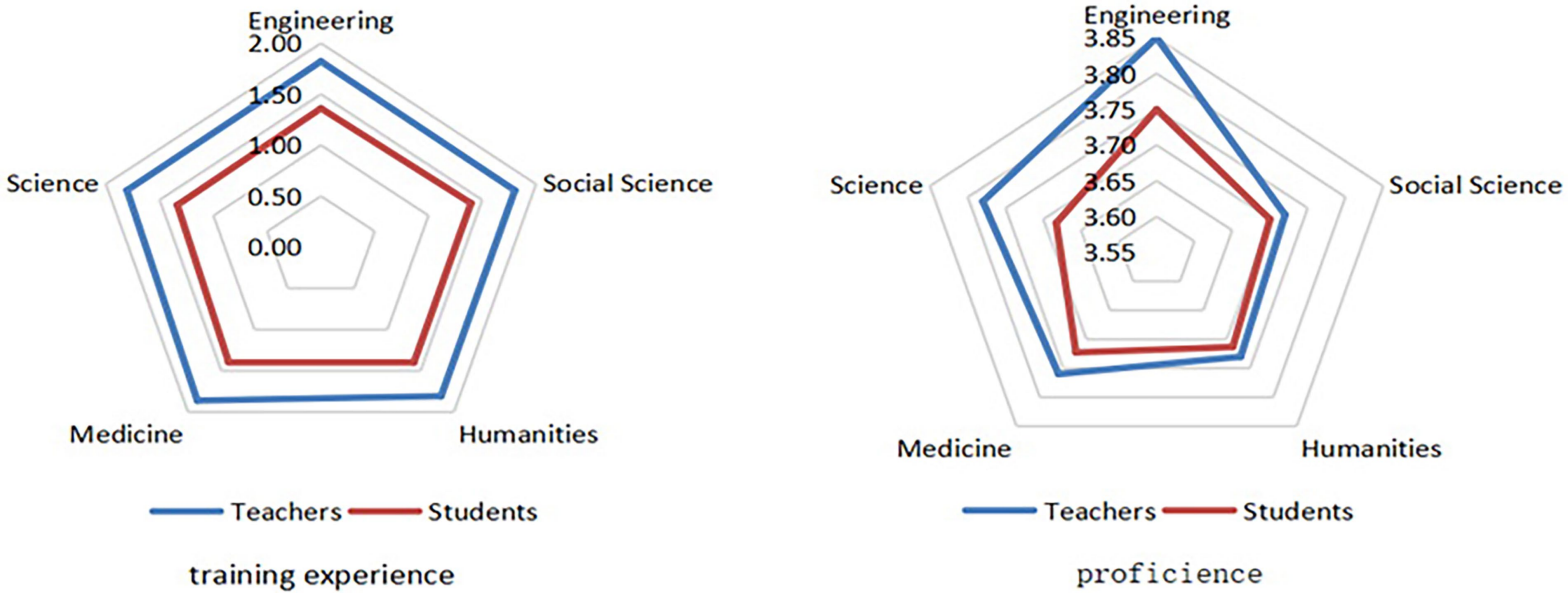
Training experience and proficiency level of online education reported by teachers and students of different disciplines.

Compared with students, teachers were more trained and generally skilled in online learning (Figure 2). This shows that Chinese colleges and universities worked to cultivate teachers’ information literacy. There was no significant difference in the training experience of teachers and students of different disciplines. In terms of proficiency, the online learning proficiency of engineering teachers and students was relatively high, illustrating their strong overall ability in the application of network tools.

#### Online service reliability

**Figure 3:**
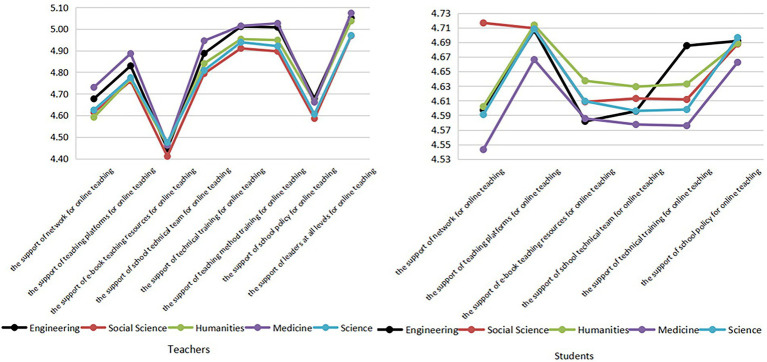
Evaluation of online service reliability by teachers and students of different disciplines.

There was little difference in teachers’ views of online service reliability among different disciplines, but large differences were found among students (Figure 3). Compared with other disciplines, medicine and social sciences students especially gave significantly higher ratings on the support of networks for online learning. Humanities students had a good experience with the basic network hardware provided by the university. On the contrary, medical students had a poor experience. Engineering students scored higher in the online learning method training provided by universities. This shows that most engineering students received relevant training on the online learning provided by universities and had a good experience. This may also be one of the reasons for the higher online learning proficiency of engineering students mentioned above.

### Disciplinary differences in learning experience

#### Teaching mode

**Figure 4:**
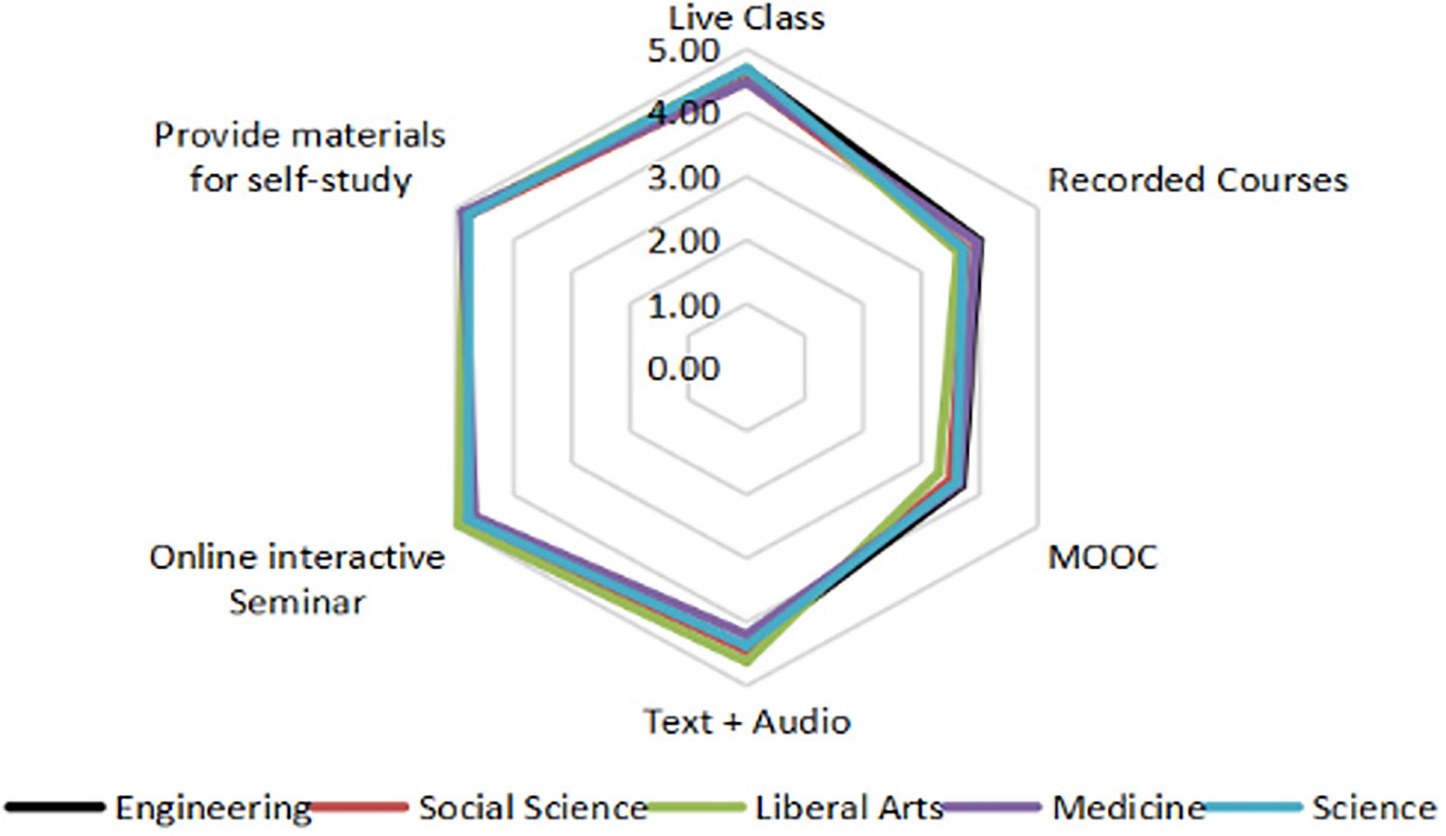
The use of online teaching modes for teachers of different disciplines.

According to Figure 4, teachers rarely used MOOCs or videos, but preferred online interactive seminars and provided students with self-study materials. There were no significant disciplinary differences in the teaching mode. This finding shows that most teachers continued to use traditional teaching modes and preferred to foster interaction.

#### Participation, assignments, and grading

**Figure 5:**
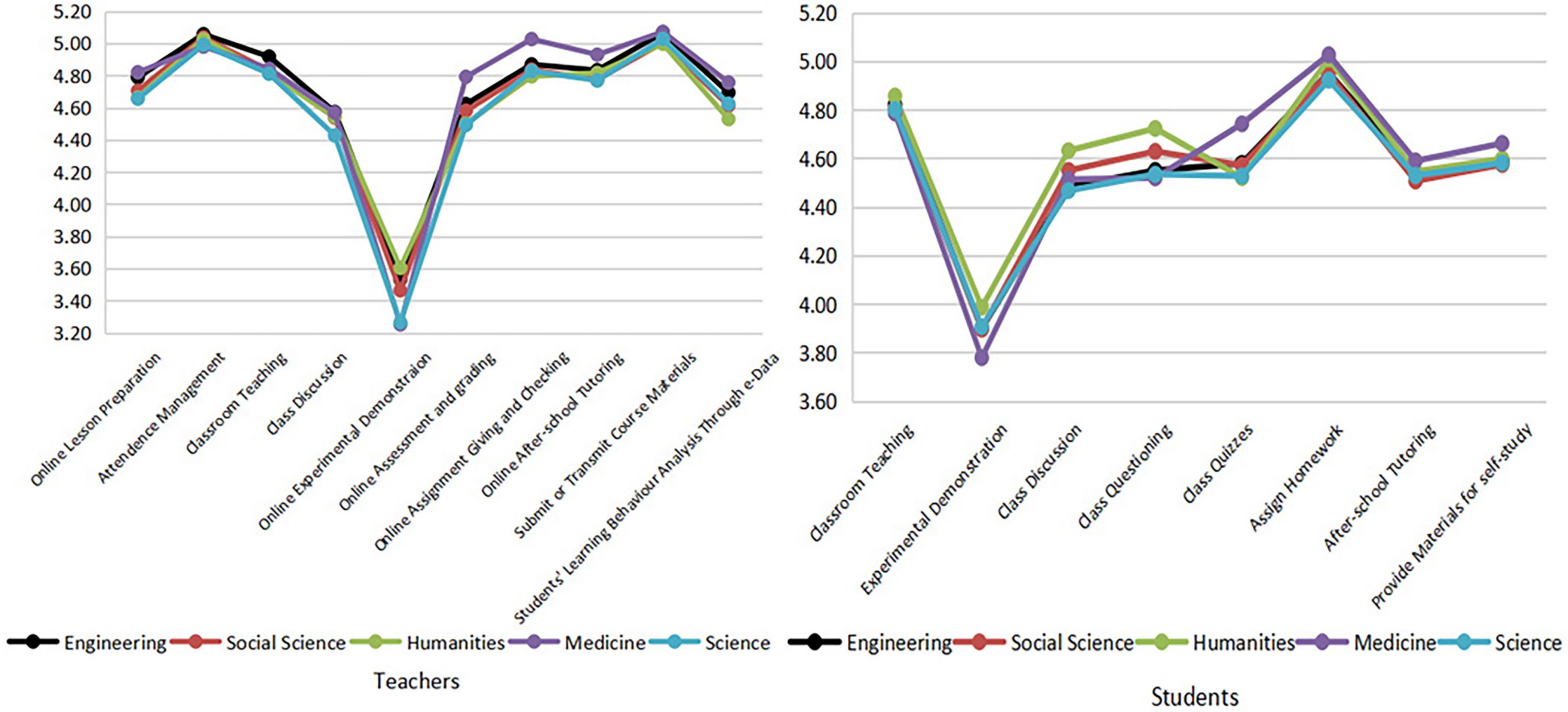
The evaluation of online pedagogical approaches by teachers and students of different disciplines.

From the perspective of teachers’ assignments and grading of different disciplines (Figure 5), medical teachers scored higher in making and grading online assignments and in giving and evaluating online examinations, which indicates that these two items were properly implemented by medical teachers. In addition, medical students gave a higher score to classroom quizzes than students in other disciplines, which is consistent with the evaluation of medical teachers, illustrating that both medical teachers and students may have chosen suitable online test methods and platform tools for their disciplines. The average scores of humanities and social sciences students in classroom questioning and discussion were higher than those of science, engineering, and medical students, which indicates that students of liberal arts and social sciences have proper classroom participation, higher enthusiasm, and better interaction.

#### Technology support

**Figure 6:**
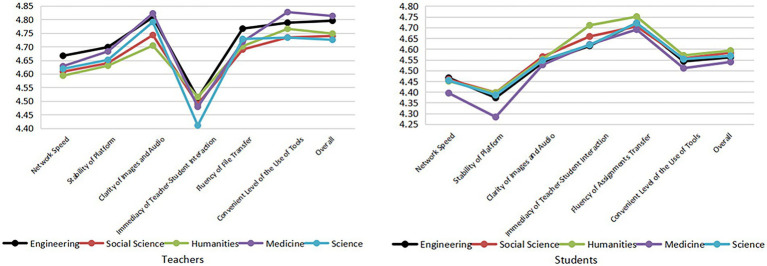
The evaluation of teaching platform technology support by teachers and students of different disciplines.

Technology support did not differ much by discipline (Figure 6). Relatively speaking, for science, there were some problems such as low frequency of classroom interaction and low learning participation of students. Medical students had a poor experience with “network speed” and “stability of platform.” The reason may be that medical courses involve experimental demonstrations and operations and therefore require high network skills. Based on Figure 5, both teachers and students expressed dissatisfaction with online experimental demonstrations. The network fluency and platform stability may be one of the reasons. Humanities students had a higher degree of real-time interaction with teachers, confirming that humanities students have a high degree of classroom participation and proper online interaction.

### Disciplinary differences in teaching feedback

#### Obstacles to online learning

**Figure 7:**
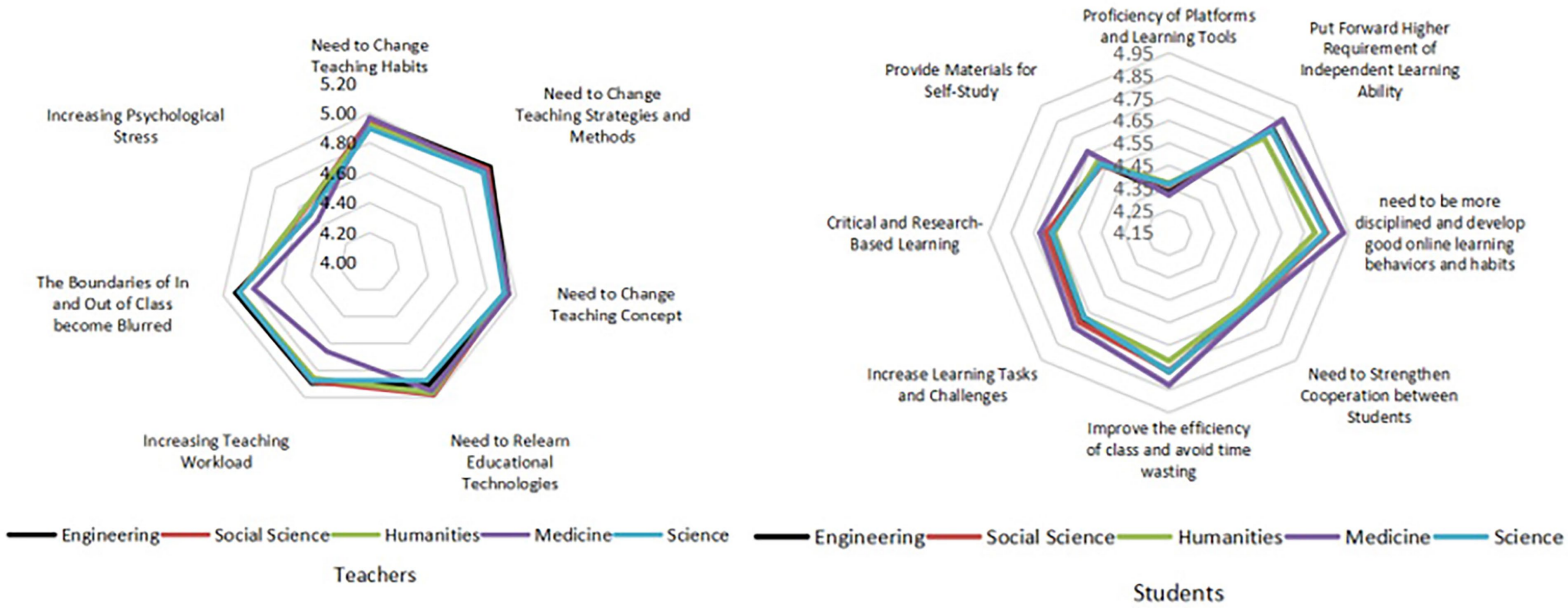
The biggest obstacle faced by teachers from different disciplines in online education.

As shown in Figure 7, most teachers considered maintaining students’ attention the biggest obstacle to online learning. This shows that students were less engaged in online learning and had a poor sense of immersive experience. Considering disciplinary differences, medical teachers marked lower scores to “making and grading online assignment” and “designing online quiz and examination,” illustrating that there are some operational difficulties in these areas.

#### Challenges to online learning

**Figure 8:**
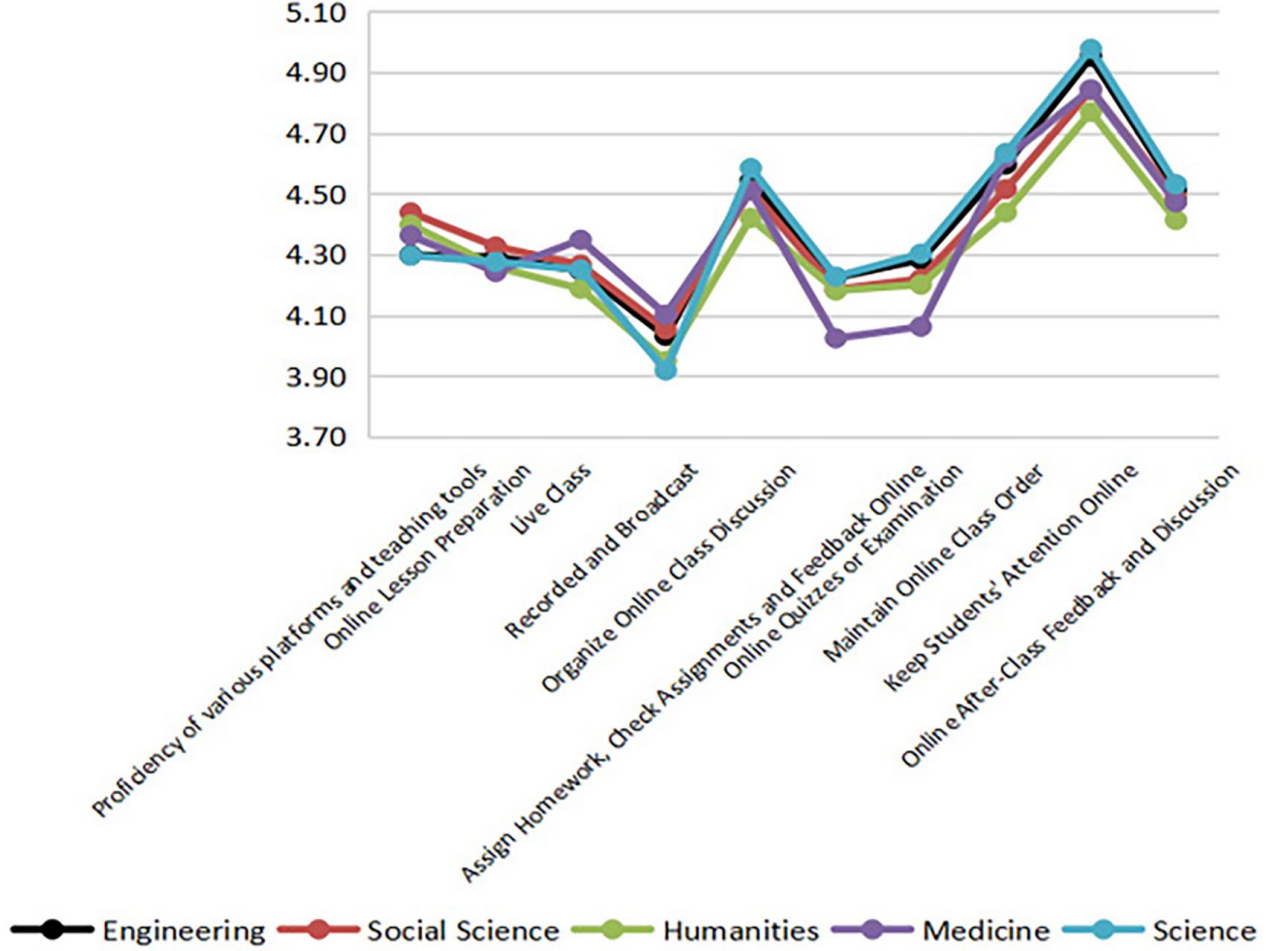
Evaluation of the challenges in online education by teachers and students of different disciplines.

Medical teachers reported that online education did not significantly increase their workload (Figure 8). This may be because medical teachers and students generally already had a relatively rich experience in online learning before the outbreak of COVID-19.

#### Factors influencing online learning

In this study, the factors influencing teachers and students of different disciplines in online learning were ranked in descending order, according to the average value. The ranking of engineering disciplines was taken as a reference group to compare with the other disciplines. As shown in Table 2, teachers in engineering, social sciences, and humanities considered the most important online learning factor to be “students’ independent learning ability,” while medical and science teachers chose “good online learning habits.” Similarly, students of four disciplines considered “students’ independent learning ability” as most important, while humanities students chose “good online learning habits.” Thus, students’ independent learning ability and good online learning habits are two important factors that affect online learning. The results also suggest that, overall, the online education situation in medicine and science is different, and it is difficult for students to develop basic online learning habits. Previous research found that the online learning mode of humanities students is mostly discussion oriented, with better interaction between teachers and students. Therefore, there may be problems such as disorder in the humanities classroom, so teachers and students of humanities believe that good online learning habit is an important factor.

**Table 2:** Evaluation of online education influencing factors by teachers of various disciplines.

Influencing factors (Teachers)	E	SS	H	M	Sc	Influencing factors (Students)	E	SS	H	M	Sc
Students’ independent learning ability	1	1	1	2	2	Students’ independent learning ability	1	1	2	1	1
Good online learning habits	3	3	2	1	1	Good online learning habits	2	2	1	2	2
Students’ active participation	2	2	3	3	3	Students’ active participation	3	3	3	3	3
Teachers’ attitude and focus on teaching	4	4	4	4	4	The teacher’s teaching strategy and teaching (demonstration) method	4	4	4	4	4
Function and stability of teaching platform	5	5	5	8	6	Students’ learning space and terminal equipment support	5	5	6	5	5
The teacher’s teaching strategy and teaching (demonstration) method	6	6	7	6	7	Teachers’ attitude and focus on teaching	6	6	7	7	6
Students’ learning space and terminal equipment support	7	7	6	5	5	Function and stability of teaching platform	7	7	5	6	7
Network speed and stability	8	9	8	10	9	Choice of course content suitable for online teaching	8	8	10	8	9
The school’s policy support for online teaching	9	8	9	7	8	The school’s policy support for online teaching	9	9	11	9	8
Choice of course content suitable for online teaching	10	10	10	9	10	Online technical support	10	10	8	11	10
Teachers’ familiarity with teaching platforms and tools	11	12	12	11	12	Provision of supporting electronic teaching resources	11	12	12	10	11
Online technical support	12	11	11	13	13	Network speed and stability	12	11	9	12	12
Provision of supporting electronic teaching resources	13	15	14	12	14	Selection of the appropriate evaluation methods	13	14	13	13	13
Teachers’ teaching space and equipment support	14	13	13	15	11	Teachers’ familiarity with teaching platforms and tools	14	13	14	14	14
Selection of appropriate evaluation methods	16	16	16	14	15	Teachers’ teaching space and equipment support	15	15	15	15	15
Students’ familiarity with teaching platforms and tools	15	14	15	16	16	Students’ familiarity with teaching platforms and tools	16	16	16	16	16
Control and maintenance of classroom teaching order	17	17	17	17	17	Control and maintain the classroom teaching order	17	17	17	17	17
Provision of a certain number of teaching assistants	18	18	18	18	18	Provision of a certain number of teaching assistants	18	18	18	18	18

E indicates Engineering; SS, Social science; H, Humanities; M, Medicine; and Sc, Science.

### Disciplinary differences in teaching improvement

#### Overall evaluation of online learning

According to teachers’ self-evaluation (Table 3), except for humanities teachers, teachers of other disciplines had strong performance on “I can submit and modify PowerPoints,” “I can assign, mark, and give feedback for online homework,” and “I can recommend various e-learning resources for students.” In addition to the first two abilities, humanities teachers had a high self-evaluation for “I can effectively organize online teaching and maintain teaching order.” As mentioned above, liberal arts classrooms may have problems with classroom order. Teachers needed to maintain order frequently and in a timely manner. Over time, they became more proficient in this ability, and their evaluation would be higher.

**Table 3:** Teachers’ online education ability of different disciplines.

Self-evaluation	Engineering		Social Sciences		Humanities		Medicine		Science	
	Average	Ranking	Average	Ranking	Average	Ranking	Average	Ranking	Average	Ranking
I can submit and modify PowerPoints.	5.24	1	5.16	1	5.14	1	5.23	1	5.2	1
I can assign, mark, and give feedback for online homework.	5.13	2	5.04	2	5.11	2	5.15	2	5.11	2
I can recommend various e-learning resources for students.	5.1	3	5	3	5.04	4	5.06	3	5.05	3
I can interact with students through various platforms.	5.06	4	4.98	5	5.04	5	4.99	4	4.99	4
I can prepare lessons effectively according to the characteristics of online teaching.	5.04	5	4.97	6	5.03	6	4.97	6	4.99	6
I can effectively organize online teaching and maintain teaching order.	5.03	6	4.99	4	5.09	3	4.97	5	4.99	5
I can carry out live broadcasts.	4.93	7	4.79	11	4.92	10	4.74	13	4.92	7
Overall, I am satisfied with my online teaching.	4.93	8	4.87	7	4.94	8	4.83	9	4.86	8
I can control the pace of teaching and avoid student fatigue.	4.91	9	4.86	8	4.97	7	4.86	8	4.85	9
I can design teaching plans suitable for online teaching.	4.91	10	4.84	9	4.9	11	4.78	11	4.82	11
I can use various tools for course testing or evaluation.	4.89	11	4.79	12	4.79	12	4.95	7	4.82	10
I can use appropriate teaching strategies to improve students’ attention.	4.86	12	4.84	10	4.94	9	4.81	10	4.79	12
I can use data to analyze and track student learning behavior.	4.79	13	4.68	13	4.69	13	4.76	12	4.72	13
I can use tools to record and broadcast.	4.77	14	4.53	14	4.47	14	4.73	14	4.6	14

**Figure 9:**
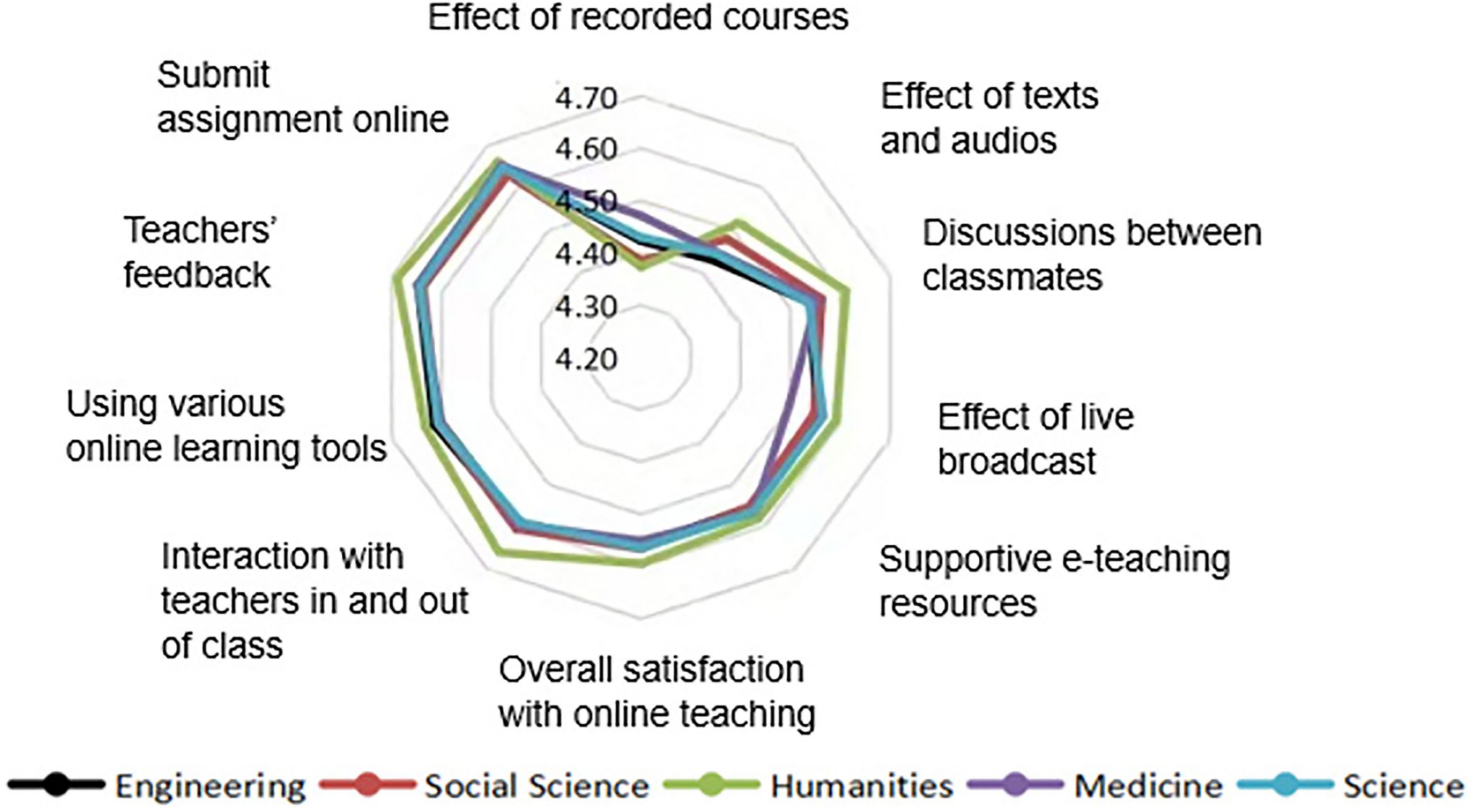
Evaluation of online learning by students of different disciplines.

From the perspective of students (Figure 9), compared with students in other disciplines, humanities students were more satisfied with discussions among classmates, interaction with teachers in and out of class, and teacher feedback on homework. This conclusion is again consistent with the previous conclusion showing good online interaction between humanities teachers and students.

#### Primary problems of online learning

As shown in Table 4, teachers generally had the same views on the primary problems of online learning, but two questions showed disciplinary differences. Teachers in engineering, social sciences, and science indicated that there were two other major problems: “students are not good at independent learning” and “students do not have good online learning habits.” Teachers in the humanities indicated two other major problems of “low network speed, unstable network connection” and “students are not good at independent learning,” while medical teachers indicated the two other major problems of “students do not have good online learning habits” and “low network speed, unstable network connection.” The construction of the online platform for humanities and medicine had problems with imperfect assurance of user experience, and humanities teachers had a worse experience regarding this problem. In addition, medical students generally had bad online learning habits, which means this discipline had an online teaching management problem, which led to a low satisfaction score. This is consistent with the conclusions above.

**Table 4:** Evaluation of the primary problems of online education by teachers and students of different disciplines.

Prime problem (Teachers)	E	SS	H	M	Sc	Prime problem (Students)	E	SS	H	M	Sc
Some of the content is not suitable for online teaching	1	1	1	1	1	Some of the content is not suitable for online teaching	1	2	3	1	2
Students are not good at independent learning	2	2	3	4	2	There is low network speed and unstable network connection	2	1	1	2	1
Students do not have good online learning habits	3	3	5	2	3	The teaching platform has imperfect functions and poor stability	3	3	2	3	3
It is difficult to maintain classroom order	4	5	7	5	4	The support of online technical service is insufficient	4	4	4	4	4
There is low network speed and unstable network connection	5	4	2	3	5	Insufficient supporting electronic teaching resources are provided	5	5	5	5	5
Students’ engagement is insufficient	6	7	8	6	6	Students are not good at independent learning	6	6	6	6	6
The teaching platform has imperfect functions and poor stability	7	6	4	7	7	Students do not have good online learning habits	7	7	7	7	7
Insufficient course-supporting electronic teaching resources are provided	8	8	6	8	9	Teaching strategies and methods for online teaching are unsuitable	8	9	9	9	9
The support of learning space and terminal equipment is insufficient	9	9	9	9	8	Support of learning space and terminal equipment is insufficient	9	8	8	8	8
The support of teaching space and equipment is insufficient	10	10	11	10	10	Students’ engagement is insufficient	10	11	11	10	10
The support of online technical service is insufficient	11	11	10	11	11	Educational evaluation methods for online teaching are unsuitable	11	10	10	11	11
Educational evaluation methods are unsuitable for online teaching	12	13	13	16	12	Support of teaching space and equipment is insufficient	12	13	13	12	14
Students are not proficient in learning platforms and tools	13	12	12	12	13	Teachers are not proficient in teaching platforms and tools	13	14	14	14	15
Teaching strategies and methods are unsuitable for online teaching	14	14	15	15	14	Policy support for online teaching is insufficient	14	15	15	13	13
Teachers are not proficient in teaching platforms and tools	15	15	14	13	16	It is difficult to maintain classroom order	15	16	16	16	16
The number of teaching assistants is insufficient	16	17	16	14	15	Students are not proficient in learning platforms and tools	16	12	12	15	12
Policy support for online teaching is insufficient	17	16	17	18	17	Teachers have insufficient attitudes and attention to online teaching	17	17	17	18	18
Teachers have insufficient attitudes and attention to online teaching	18	18	18	17	18	The number of teaching assistants is insufficient	18	18	18	17	17

E indicates Engineering; SS, Social science; H, Humanities; M, Medicine; and Sc, Science.

From the perspective of students, engineering and medical students indicated the primary problem of online education was that “some of the content is not suitable for online teaching.” It was shown above that teachers preferred more interactive teaching modes, such as live classes. Therefore, combined with this conclusion, it was found that engineering and medical teachers just “transfer” the traditional teaching content to an online platform without considering whether the content was suitable for online teaching. Social sciences, liberal arts, and science students viewed the “speed and stability of the network” as the primary problem in online education, which indicated the online education technology needed to be improved.

#### Continuation of online learning

**Figure 10:**
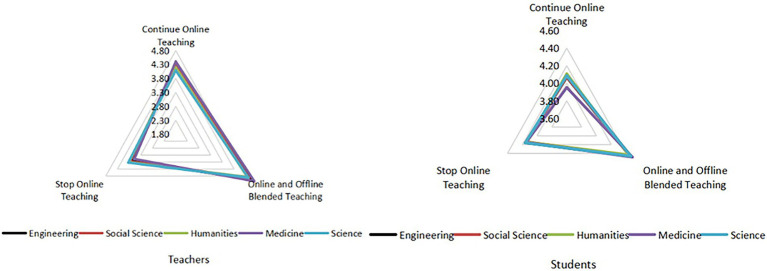
Views on the continuation of online education from teachers and students of different disciplines.

The average score of medical students on the continuation of online education was significantly lower than that of other disciplines (Figure 10), which means that most medical students do not support a pure online education mode, and the medical teaching content is not suitable for pure online education.

#### Suggestions for the improvement of online learning

As shown in Table 5, teachers of engineering, social sciences, and humanities indicated that to improve online learning in the post-COVID-19 era requires efforts to “improve students’ ability of independent learning,” “guide students to form a good habit of online learning,” and “select suitable teaching content for online teaching.” This finding verifies the results on problems with current online education practice, such as students’ poor independent learning ability and online learning habits, teachers’ poor online teaching adaptability, and a low matching degree between related resources of online teaching and actual teaching practice. Moreover, science teachers indicated there was a need to “increase the students’ participation” to improve online education, which shows the low participation of science students. This is consistent with the previous conclusion.

**Table 5:** Evaluation of suggestions for the improvement of online education by teachers and students of different disciplines.

Suggestions for improvement (Teacher)	E	SS	H	M	Sc	Suggestions for improvement (Student)	E	SS	H	M	Sc
Improve students’ ability of independent learning	1	1	1	1	1	Select teaching content suitable for online teaching	1	2	4	1	2
Guide students to form good habits in online learning	2	2	2	2	2	Improve platform function and stability	2	1	2	2	1
Increase students’ participation	4	4	6	4	3	Improve network speed and stability	4	3	1	6	3
Select teaching content suitable for online teaching	3	3	3	3	4	Improve support for online technical services	3	4	3	3	4
Improve platform function and stability	5	5	4	6	5	Enrich the course-supporting electronic teaching resources	5	5	5	4	7
Enrich course-supporting electronic teaching resources	6	6	5	5	6	Guide students to form good habits in online learning	6	6	6	5	5
Improve the teaching space environment and equipment of teachers	7	10	9	10	7	Improve students’ ability of independent learning	7	7	7	7	6
Improve network speed and stability	8	8	7	9	9	Change teaching strategies and methods	8	8	8	8	8
Increase policy support for online teaching	9	9	11	8	11	Improve the learning space environment and the support of equipment	9	10	10	10	9
Improve support for online technical services	10	7	8	7	8	Help teachers put more effort into teaching	10	9	9	9	10
Improve the learning space environment and the support of equipment	11	11	10	11	10	Increase policy support for online teaching	11	11	11	12	11
Change teaching strategies and methods	12	13	13	12	13	Increase students’ participation	12	12	12	11	12
Improve classroom order management	13	14	14	14	14	Improve the teaching space environment and equipment of teachers	13	13	13	13	13
Improve the guidance given to students on the use of teaching platforms and tools	14	12	12	13	12	Improve classroom order management	14	15	15	14	14
Reform educational evaluation methods	15	15	16	15	15	Improve the guidance given to students on the use of teaching platforms and tools	15	14	14	15	15
Helpe teachers put more effort into teaching	16	17	17	16	16	Improve online teaching-related training	16	16	16	16	16
Improve online teaching-related training	17	16	15	17	17	Reform educational evaluation methods	17	18	18	18	18
Provide teaching assistants	18	18	18	18	18	Provide teaching assistants	18	17	17	17	17

E indicates Engineering; SS, Social science; H, Humanities; M, Medicine; and Sc, Science.

Generally, students had similar opinions on suggestions to improve online education, but there were also some different voices. Engineering and medical students indicated that the primary improvement direction should be to “select teaching content suitable for online teaching,” while social sciences and science students indicated that it should be to “improve platform function and stability,” and humanities students indicated that it should be to “increase network speed and stability.” Difference in students’ recommendations are related to the different training aims of different disciplines. On the other hand, they also highlight characteristics of online learning during the COVID-19 pandemic.

From the data of descriptive statistics, it can be found that there are differences between teachers and students in different disciplines in teaching and learning preparation, experience, feedback and improvement. In order to test whether there are significant differences between teachers and students in different disciplines in the four dimensions, the study conducted the analysis of variance on the online educational situation of teachers and students in each discipline. According to the results of the variance homogeneity test, the variance differences of other variables were all significant at the 0.05 level, except that the variance differences of teachers’ online teaching after the outbreak of COVID-19 were not significant (Tables 6, 7).

**Table 6:** Difference test of teachers’ online education of different disciplines.

Dimensions	Variables		SS	df	MS	F
Teaching preparation	Implementation of online education before the outbreak of COVID-19	BG	11.009	4	2.75225	16.972^***^
		WG	2220.063	13,690	0.1621668	
		Sum	2231.072	13,694		
	Implementation of online education after the outbreak of COVID-19	BG	0.057	4	0.01425	4.083
		WG	47.775	13,690	0.0034898	
		Sum	47.832	13,694		
	Training experience of online education	BG	9.279	4	2.31975	15.234^***^
		WG	2084.675	13,690	0.1522772	
		Sum	2093.954	13,694		
	Proficiency level of online education	BG	94.354	4	23.5885	51.423^***^
		WG	6279.809	13,690	0.458715	
		Sum	6374.163	13,694		
	Online service reliability	BG	30.557	4	7.63925	13.332^***^
		WG	7844.659	13,690	0.5730211	
		Sum	7875.216	13,694		
Teaching experience	Use of online teaching modes	BG	240.710	4	60.1775	42.912^***^
		WG	19198.326	13,690	1.4023613	
		Sum	19439.040	13,694		
	Online pedagogical approaches	BG	53.395	4	13.34875	18.209^***^
		WG	10035.891	13,690	0.7330819	
		Sum	10089.286	13,694		
	Technology support of teaching platform	BG	26.284	4	6.571	11.686^*^
		WG	7697.825	13,690	0.5622955	
		Sum	7724.109	13,694		
Teaching feedback	Obstacles to online education	BG	162.046	4	40.5115	31.874^***^
		WG	17399.680	13,690	1.2709774	
		Sum	17561.726	13,694		
	Challenges to online education	BG	32.311	4	8.07775	9.632^***^
		WG	11481.143	13,690	0.8386518	
		Sum	11513.454	13,694		
	Factors influencing online education	BG	39.229	4	9.80725	16.332^**^
		WG	8220.539	13,690	0.6004776	
		Sum	8259.768	13,694		
Teaching improvement	Overall evaluation of online education	BG	36.845	4	9.21125	20.522^***^
		WG	6144.856	13,690	0.4488573	
		Sum	6181.701	13,694		
	Primary problems of online education	BG	38.442	4	9.6105	8.076^***^
		WG	16292.026	13,690	1.1900676	
		Sum	16330.468	13,694		
	Continuation of online education	BG	126.072	4	31.518	24.637^***^
		WG	17513.286	13,690	1.2792758	
		Sum	17639.358	13,694		
	Suggestions for the improvement of online education	BG	57.499	4	14.37475	19.097^***^
		WG	10305.027	13,690	0.7527412	
		Sum	10362.526	13,694		

SS indicates Sum of Squares; MS, Mean Squares; df, degree of freedom; F, F test; BG, Between Groups; and WG, Within Groups.

* *p* < 0.05;

** *p* < 0.01;

*** *p* < 0.001.

**Table 7:** Difference test of students’ online learning of different disciplines.

Dimensions	Variables		SS	df	MS	F
Learning preparation	Implementation of online learning before the outbreak of COVID-19	BG	211.583	4	52.89575	213.522^**^
		WG	62409.067	251,924	0.24773	
		Sum	62620.650	251,928		
	Implementation of online learning after the outbreak of COVID-19	BG	3.180	4	0.795	93.448^***^
		WG	2143.215	251,924	0.008507	
		Sum	2146.395	251,928		
	Training experience of online learning	BG	212.353	4	53.08825	226.697^***^
		WG	58995.952	251,924	0.234182	
		Sum	59208.300	251,928		
	Proficiency level of online learning	BG	513.291	4	128.3228	211.337^*^
		WG	152967.149	251,924	0.607196	
		Sum	153480.440	251,928		
	Online service reliability	BG	268.863	4	67.21575	86.21^***^
		WG	196418.638	251,924	0.779674	
		Sum	196687.500	251,928		
Learning experience	Use of online learning modes	BG	1181.262	4	295.3155	328.778^***^
		WG	226283.700	251,924	0.898222	
		Sum	227464.960	251,928		
	Online pedagogical approaches	BG	522.630	4	130.6575	177.374^***^
		WG	185573.042	251,924	0.736623	
		Sum	186095.670	251,928		
	Technology support of learning platform	BG	292.291	4	73.07275	100.427^***^
		WG	183304.572	251,924	0.727619	
		Sum	183596.860	251,928		
Learning feedback	Challenges to online learning	BG	440.504	4	110.126	102.033^**^
		WG	271905.928	251,924	1.079317	
		Sum	272346.400	251,928		
	Factors influencing online learning	BG	517.806	4	129.4515	152.37^***^
		WG	214031.321	251,924	0.849587	
		Sum	214549.130	251,928		
Learning improvement	Overall evaluation of online learning	BG	283.080	4	70.77	81.195^***^
		WG	219579.191	251,924	0.871609	
		Sum	219862.270	251,928		
	Primary problems of online learning	BG	399.408	4	99.852	89.035^***^
		WG	282530.548	251,924	1.121491	
		Sum	282929.960	251,928		
	Continuation of online learning	BG	612.844	4	153.211	105.982^***^
		WG	364187.851	251,924	1.445626	
		Sum	364800.700	251,928		
	Suggestions for the improvement of online learning	BG	574.074	4	143.5185	157.416^***^
		WG	229682.559	251,924	0.911714	
		Sum	230256.630	251,928		

SS indicates Sum of Squares; MS, Mean Squares; df, degree of freedom; *F*, F test; BG, Between Groups; and WG, Within Groups.

* *p* < 0.05;

** *p* < 0.01;

*** *p* < 0.001.

## Discussion

The results showed that teachers and students of different disciplines had varying views on the four research dimensions of this study, answering Question 1. In this section, the study’s findings are compared with previous research results to discuss the research question.

### Obvious disciplinary differences in the preparation of teachers and students and in support from universities

Since COVID-19 was an unexpected public crisis, large-scale online education was an “encounter” without sufficient mental preparation (Wu and Li, 2020). About 80% of college teachers and 60% of college students had not participated in online learning before (Center for Teaching and Learning Development of Xiamen University, 2020a,b). However, medical teachers and students had a relatively rich experience in online learning before the outbreak of COVID-19. With the development of large-scale online learning, the number of teachers and students using online education increased significantly, with similar participation across the disciplines. Furthermore, an online education mode was fully achieved in a very short time.

In terms of disciplinary differences, the online learning proficiency in engineering was the highest, followed closely by science and medicine, both of which had higher scores than liberal arts and social sciences. Therefore, Questions 2, which asked if teachers and students of five disciplines groups have different levels of proficiency in the online education technology, was answered. According to Wu et al. (2020), the reason is that learning in science, engineering, agriculture, and medicine often involves experiments and other practical methods. What is more, the communication and interaction between teachers and students of these disciplines may rely more on software and terminal equipment, which leads to a better grasp of technology.

Another explanation for the findings may relate to the construction of “New Engineering Disciplines,” with the goal of cultivating talent and developing intelligent manufacturing in China (Yu et al., 2019). Although engineering teachers and students had less online education experience than medical teachers and students before the outbreak of COVID-19, their in-depth online education experience was better than that of other disciplines due to the promotion of the construction of “New Engineering Disciplines.”

From the perspective of the online service reliability offered by universities, the attitudes of teachers did not vary much by discipline, but certain differences were evident among students. Compared with students in other disciplines, students from medical and social sciences disciplines had a better experience with the network reliability (both hardware and software) offered by universities. Engineering students gave better ratings to the online education methods provided by universities compared with students in other disciplines.

### Minor disciplinary differences in the teaching process and the evaluation of technical support

Teaching mode determines the design and implementation of the teaching process to some extent and also affects the choice of teaching platform. The study showed minor disciplinary differences exist in the teaching process among teachers, which answered Question 3. Teachers preferred to organize online interactive seminars and to provide self-study materials, rather than use MOOCs or recordings.

In terms of pedagogical approaches, the differences between teachers and students in different disciplines were not significant. Relatively speaking, medical teachers had higher scores for online assignment making, checking, testing, and grading. At the same time, humanities and social sciences students had a better experience with classroom questioning and discussions, which answered Question 4. Medical students had a better experience with classroom quizzes. Some medical universities have established evaluation systems to optimize the evaluation process (Zhang et al., 2020).

The progress of teaching activities is inseparable from the support of the technical platform. This study showed that all teachers, especially science teachers, considered the lack of timely teacher-student interaction as a problem. On one hand, due to the current imperfect construction of the network communication platform, the large amount of people using the network at the same time can easily lead to network congestion, frequent internet lag, and a high rate of lost connection during peak hours (Huang et al., 2020), which delays the immediacy of teacher-student interaction to some extent. On the other hand, science, as a “pure hard science” discipline group (Becher and Trowler, 2008) has rigorous structure, high consistency between course content and professional judgment, and emphasis on truths, principles, and concepts teaching (Braxton, 1995; Braxton and Hargens, 1996). Therefore, science classes have less teacher-student interactions but mainly rely on teacher’s introduction and explanation. Due to the lower requirements for experimentation compared with medicine, the experimental classes of science disciplines during the COVID-19 have always been replaced with watching recorded videos. The double absence of teacher-student interaction in both theoretical and experimental classes leads to dissatisfaction in this discipline.

As to students, it was found that medical students had a poor experience with “network speed” and “platform stability.” Medical students have higher requirements for platform stability and network fluency as the medical courses rely on a large number of resources for demonstration and real-time explanation (Chu et al., 2020). However, there is a gap between the reality of network quality and the teaching demand, which leads to an unsatisfactory experience for medical students. In addition, it was found that humanities teachers and students had a better experience with the immediacy of teacher-student interaction since all of the humanities disciplines are “pure soft science” (Becher and Trowler, 2008). Disciplines of “pure soft science” pay more attention to teacher-student interaction and apply a student-centered learning mode (Hativa, 1997). This is why teacher-student interaction in this discipline is still better than that of other disciplines even if the teaching activities are “transplanted” from a traditional classroom to an online classroom. In addition, disciplines of “pure soft science” focus on the cultivation of students’ oral and written expression (Braxton et al., 1998). Therefore, the interaction between teachers and students of this discipline is mostly conducted by online communications such as instant messaging, voice calls, etc. Compared with medicine and engineering, which need to do experiments, the soft science disciplines do not have higher requirements for platform technology. Many platforms can meet their requirements. Therefore, the immediacy of teacher-student interaction can be effectively secured.

### Obvious disciplinary differences in the constraints of online education between teachers and students

Compared with traditional teaching, the implementation of high-quality online education involves multidirectional information transmission between teachers and students and requires a stable terminal and network environment and instant transfer of messages and information (Yu, 2020). The environment is undoubtedly difficult for teachers and students who have little online educational experience. Teachers generally believe that “maintaining students’ attention online” is the biggest problem. This is not only a problem in colleges and universities, but also a problem faced by many students and teachers of different educational periods. The reasons include not only the subjective factors, but also the school management’s lack of scientific teaching plans (Wang et al., 2020).

Specifically, from the perspective of disciplinary differences, this study found that medical teachers had significantly more difficulties in “online assignment and making homework” and “online tests or examinations” than teachers in other disciplines. The medicine examination is mainly divided into theoretical and practical parts (Gui et al., 2020). However, the practical examination parts such as medical experiments cannot be carried out in a traditional laboratory due to the impact of COVID-19, so they can only rely on virtual simulation experiment technology. Nowadays, the comprehensive platform of virtual simulation experimental teaching in China still has some problems, such as a shortage of funds, limited technical personnel, and an insufficient sharing system (Gong et al., 2019). The VR and AR equipment involved in real scene operation is too expensive that not each university can afford them in their teaching process. Under this situation, most medical teachers can only examine students’ actual practical ability through a “screen.” This “virtual surgery” not only seriously affects medical students’ learning experience, but also makes it difficult for teachers to know the actual ability of students and to judge or guide students properly. As a result, medical teachers believe that assignment-and examination-related problems are the most difficult.

However, medical teachers have done quite well in addressing these two problems. Due to the balanced and random proportion of the disciplines selected and investigated in this study, apart from sample factors, we conclude that the reason for this contradictory opinion may be that teachers and students in medicine generally had rich online education experiences before the pandemic, so there is a certain “presupposition” for some of the problems existing within the discipline. Some studies have shown that a group of Chinese domestic medical colleges have carried out a series of preparations at the beginning of online education and set up a teaching quality work team to analyze and solve problems in the practice stage (Chi et al., 2020; Yuan et al., 2020). Therefore, the preparatory work based on presupposition, coupled with rich practical experience, has enabled medical teachers to successfully overcome difficulties.

Focusing on the views of teachers and students of different disciplines on the primary factors affecting the effect of online education, the findings showed that engineering, social sciences, and humanities teachers believed “students’ independent learning ability” as the primary factor, while medical and science teachers considered the primary factor as “good online learning habit.” From the perspective of students, humanities students thought the primary factor is “good online learning habits,” while other students thought it is “students’ independent learning ability.” Both teachers and students believed that students’ independent learning ability and good online learning habits are important factors affecting the effect of online education. Therefore, the quality of students’ autonomous learning is the primary factor that teachers and students generally believed affects the online education effect. This is consistent with the conclusion of previous studies on self-directed learning and the effectiveness of online education (Artino and Stephens, 2009; Spector, 2015).

Focusing on the attitudes of medical and science teachers and liberal arts students through follow-up interviews showed that although the three groups all believed that “good online learning habits” was the primary factor affecting the effect of online education, the reasons behind the choice were different. Medical and science teachers held this view because of their students’ poor participation, frequent absenteeism, and fake online status. For humanities students, the internet lag or the loss of connection led to chaos in classroom discussions, thus affecting the effect of online learning. Therefore, whether from the perspective of phenomenon or attribution, teachers and students of different disciplines had specific needs, suggesting that the management of online education should provide targeted services according to the requirements and characteristics of disciplines.

### Minor disciplinary differences between teachers and students in views on the future of online education

Although there was consistency, slight differences still existed in the attitudes of teachers and students of different disciplines on the improvements and future directions of online education, which answered Question 5. As to teaching mode in the post-COVID-19 era, teachers and students supported the adoption of an online + offline teaching mode, such as “blended teaching.” Although online education presents some advantages over traditional teaching, those advantages are not sufficient for the national popularization of pure online education in a very short time. Some problems still exist in China, such as platform technical problems, the insufficient information literacy and adaptability of users, and the equity issue caused by significant differences by regions, educational stages and schools. Among the disciplines, medical students are less supportive of pure online education than students of other disciplines. This is consistent with the results of a study on online education practice for medical students (Wang et al., 2020).

Focusing on teachers’ self-evaluation of abilities in online education, teachers generally had a stronger performance on “I can submit and modify PowerPoints,” “I can assign, mark and give feedback on homework online,” and “I can recommend online learning resources for students.” The essence of online education is the online interaction of multiple subjects (Cheng, 2013) based on the interactive mode in the online learning process (Moore, 1989). Yet the current interactive mode that teachers are good at does not emphasize real-time human-to-human or human-computer feedback. In addition to the first two abilities, humanities teachers had a high self-evaluation of the ability that “I can effectively organize online education and maintain teaching order.” Although this approach also involves human-to-human interaction, it requires immediate feedback between teachers and students, which is relatively difficult. The online interaction level of humanities teachers was higher. Because of this, humanities students were also relatively more satisfied than students in other disciplines, in terms of mutual discussions among students, interaction with teachers in and out of class, and teachers’ feedback on homework.

Teachers and students have successfully completed the transition from traditional teaching to online education confronted with the challenge of large-scale online education. At the same time, teachers and students also held certain opinions about existing problems. First, teachers generally believed the primary problem of online education was that “some teaching content is not suitable for online teaching.” The reason was that some resources of recorded courses and MOOCs came from universities’ courses before the pandemic, so the actual teaching condition during the pandemic was not considered. Besides, some researchers have indicated that the appropriateness of the recorded courses used by some universities needed to be considered (Yu, 2020). Some live-streaming courses also had the problem of teaching content unsuitable for online education (Wu et al., 2020), which shows the inconsistency between teachers’ cognition and actual practice.

## Limitations

This study had a number of limitations. For instance, it only used the average value calculating as statistical analysis as a research method. Other statistical research methods could be applied for in-depth and accurate analysis in subsequent studies. Moreover, the reasons for differences in the online education experience of teachers and students in a certain dimension, especially the reason for the differences in the effect of and satisfaction with online education, can be selected as a direction for further research. This study did not survey all Chinese university teachers and students who participate in online education, so the macro data does not fully reflect all characteristics. Since relevant data on this large-scale online education practice were not kept before the outbreak of COVID-19, the precise direction of improvement in the post-COVID-19 era still needs to be monitored and analyzed. Large-scale online education practice was not common, as it occurred because of the outbreak of COVID-19. Thus, it will be valuable to systematically and comprehensively summarize and rethink everything it brought to us and then improve the informatization of education and change the teaching mode.

## Conclusion

As the post-COVID-19 era is coming, education will gradually return to traditional methods. How to combine information technology and traditional education teaching successfully based on disciplinary characteristics will be an important follow-up problem.

This study described the differences between teachers and students of different disciplines in teaching preparation, teaching experience, teaching feedback, and teaching improvement. The findings showed that teachers and students were swamped by the outbreak of COVID-19 with insufficient teaching preparation and experience. There were significant disciplinary differences between teachers and students in subjective preparation and the objective support of the school. In terms of teaching experience, the study found no significant disciplinary differences between teachers and students in this dimension. Specifically, teachers generally preferred teaching modes with high interactive frequency, such as live broadcast. There were minor disciplinary differences in pedagogical approaches and the application of platform technology. In the teaching feedback stage, there were some differences in the details for the difficulties, challenges, and attributions of online education, but the trend was overall consistent. Finally, in terms of teaching improvement, although teachers and students had different expectations for the future direction of online education improvement due to disciplinary differences, the supportive attitude toward the application of online education and an “blended teaching mode” in the post-COVID-19 era remains the same.

Although the pandemic has had a great impact on the teaching conditions of Chinese universities, it has also forced universities to apply comprehensive online teaching on campus. This kind of ERT will be not temporary, but can promote the iterative update of teaching skills in universities. With this vision, this research investigated teachers and students’ online experience, analyzed the differences in different disciplines, and drew the conclusions for educators, researchers, and policy makers to improve the online education. For educators, this research mainly aims at making them understand the real experiences and demands of students in different disciplines in the process of online learning. In this way, they will realize that they should focus on the planning and design of course contents in order to improve the quality of online teaching. For researchers, the research can provide them with data for reference. It makes more scholars realize that the role of teachers in online teaching has changed. Teachers should be students’ facilitators and supporters based on the “student-centered” concept. For policy makers, this study provides a reliable basis for formulating educational technology policies. It also calls for support of students’ online learning conditions and skills.

## Data availability statement

The original contributions presented in the study are included in the article/supplementary material, further inquiries can be directed to the corresponding author.

## Ethics statement

Ethical review and approval was not required for the study on human participants in accordance with the local legislation and institutional requirements. Written informed consent for participation was not required for this study in accordance with the national legislation and the institutional requirements.

## Author contributions

All authors listed have made a substantial, direct, and intellectual contribution to the work and approved it for publication.

## Funding

This work has been supported by the Humanities and Social Sciences Youth Foundation, Ministry of Education of the China (Project No. 20YJC880060, Project name: “A Study on the Occupational Status Attainment Mechanism and Promotion Strategies of Rural College Students in the Central and Western Regions in the Context of Rural Revitalization”) and by the Humanities and Social Sciences Youth Foundation, Ministry of Education of the China (Project No. 21YJC880011, Project name: “Research on the System Construction and Operating Mechanism of College Online Teaching Alliance in Germany”). Henan Province College Philosophy and Social Science Innovation Team Support Program (Project No. 2021-CXTD-05).

## Conflict of interest

The authors declare that the research was conducted in the absence of any commercial or financial relationships that could be construed as a potential conflict of interest.

## Publisher’s note

All claims expressed in this article are solely those of the authors and do not necessarily represent those of their affiliated organizations, or those of the publisher, the editors and the reviewers. Any product that may be evaluated in this article, or claim that may be made by its manufacturer, is not guaranteed or endorsed by the publisher.
